# A Non-parametric Analysis of Gender Differences With the Impacts of Applied Behavior Analysis on Autistic Individuals

**DOI:** 10.7759/cureus.60794

**Published:** 2024-05-21

**Authors:** Tami Peterson, Jessica Dodson, Robert Sherwin, Frederick Strale,

**Affiliations:** 1 Hyperbaric Oxygen Therapy, The Oxford Center, Brighton, USA; 2 Applied Behavior Analysis, The Oxford Center, Brighton, USA; 3 Hyperbaric Oxygen Therapy, Wayne State University School of Medicine, Detroit, USA; 4 Biostatistics, The Oxford Center, Brighton, USA

**Keywords:** gender-differences, autism spectrum disorder (asd), applied behavior analysis, cumulative target behaviors, treatment effect

## Abstract

Introduction

Considering the scarcity of research that directly investigates the differences between genders in their response to applied behavior analysis (ABA) therapy for individuals diagnosed with autism spectrum disorder (ASD), the objective of this study is twofold. First, it aims to reinforce the male-to-female ratio reported in existing scientific literature, thereby contributing to a broader understanding of gender distribution in ABA therapy for ASD. Second, it seeks to identify gender-based differences in aggregate target behaviors at various time intervals using three distinct datasets. The goal is to determine if gender influences the effectiveness of ABA therapy for ASD, which could inform future therapeutic strategies. Ultimately, this study strives to enhance our understanding of gender disparities in ABA therapy response among ASD individuals and hopes to improve therapeutic outcomes for all, regardless of gender.

Materials and methods

Three to five behavioral technicians per child collected daily general target mastery data for 263 individuals with autism. This data was gathered using a large N design through retrospective chart reviews within the “Catalyst” tracking software (DataFinch Technologies, Atlanta, USA). Three separate datasets were collected from June 7, 2023 to January 7, 2024. Behavior analysts employed a mixed model of discrete trial training, mass trials, and naturalistic environment treatment over seven months. General target mastery data was assembled for 259 children and four adults, with seven data instances missing. Descriptive statistics encompassed central tendency and dispersion measures, including the data distribution's mean, standard deviation, median, and range. Non-parametric inferential analysis was performed with the Mann-Whitney U test.

Results

Mann-Whitney U computations resulted in non-significant gender differences on all gender comparisons for the three datasets covering the seven-month timeframe.

Dataset #1: Time 1-(U=727.5, p=0.846, ή2=0.0002, Time 2-(U=736, p=0.910, ή2=0.00005), Time 3-(U=687.5, p=0.569, ή2=0.001)

Dataset #2: Time 1-(U=781, p=0.383, ή2=0.003), Time 2-(U=819.5, p=0.585, ή2=0.001), Time 3-(U=825, p=0.618, ή2=0.001)

Dataset #3: Time 1-(U=395, p=0.198, ή2=0.007), Time 2-(U=373.5, p=0.365, ή2 =0.003), Time 3-(U=363, p=0.471, ή2=0.002), Time 4-(U=366.5, p=0.436, ή2 =0.003), Time 5-(U=371, p=0.391, ή2=0.003), Time 6-(U=394, p=0.208, ή2=0.007), Time 7-(U=373, p=0.373, ή2=0.003), Time 8-(U=371.5, p=0.387, ή2=0.003), Time 9-(U=464.5, p=0.512, ή2=0.002), Time 10-(U=356.5, p=0.546, ή2=0.002), Time 11-(U=357.5, p=0.535, ή2=0.002), Time 12-(U=350.5, p=0.346, ή2=0.004)

Conclusions

This study suggests no significant gender differences in response to ABA therapy among individuals with autism, indicating its potential effectiveness for both genders. However, these findings should be interpreted cautiously due to statistical uncertainties reflected in the broad confidence intervals as they hint at possible substantial gender differences. Further research, including an extension study, must confirm these results and understand potential gender nuances in ABA therapy response. This could help tailor more effective, personalized therapeutic strategies for individuals with autism.

## Introduction

Scientific progress hinges on the principles of replicability and reproducibility and is sometimes overshadowed by new models. These investigations enhance our trust in the validity of prior findings and are vital for driving science forward [1]. As per data from the Center for Disease Control (CDC), it is projected that autism spectrum disorder (ASD) affects one in every 36 children. This condition is seen across all socioeconomic, ethnic, and racial demographics. Notably, the occurrence of ASD is nearly four times more common in boys than in girls [2,3]. Applied behavior analysis (ABA) therapy is universally recognized as the gold standard in the treatment of ASD. This acknowledgment is rooted in extensive research and significant supporting evidence [4-7].

These verifications are outlined in many studies. Researchers conducted a meta-analysis of 14 randomized control trials involving 555 participants, showing that ABA had a moderate to high impact and yielded significant benefits for children with ASD [8].

A systematic review of 29 studies found that ABA programs were moderate to highly effective, providing considerable benefits to children with ASD [9]. In a randomized controlled trial that included 28 children with autism, the most significant changes in intelligence scores were observed in participants in the comprehensive ABA group [10].

A systematic review and meta-analysis of 25 studies were conducted to evaluate the clinical effectiveness of early intensive ABA-based interventions for children with autism. The researchers noted significant heterogeneity, with effects differing widely across studies. They highlighted that due to insufficient data, the impact of the intervention on autism symptom severity, language development, and school placement remains indeterminate. The long-term effects are also uncertain due to a lack of follow-up data [11].

An evaluation of the effects of ABA on developmental outcomes in children with ASD and the related parental stress was carried out based on a review of 11 studies involving 632 participants. Compared to standard treatment or minimal to no treatment, comprehensive ABA-based interventions exhibited medium effects on intellectual functioning and adaptive behavior. However, there were no improvements beyond the control groups in language abilities, symptom severity, or parental stress [12].

A comprehensive search of seven online databases identified peer-reviewed English studies exploring the impact of ABA on health outcomes. The researchers classified the measured ABA outcomes into eight categories: cognitive, language, social/communication, problem behavior, adaptive behavior, emotional, autism symptoms, and quality of life [13].

A separate group of researchers underscored numerous meta-analyses, systematic reviews, and cost-benefit analyses that confirmed the effectiveness of ABA-based interventions for individuals with autism. However, they pointed out an “efficacy-effectiveness gap” due to individual heterogeneity, lower compliance levels, general medical presentation compared to specialist settings, less standardized and monitored treatments, and cost pressures [14].

Despite the robust evidence supporting its effectiveness, the adoption of evidence-based procedures remains low. Misunderstandings and misconceptions about ABA are common, and the challenges in determining suitable research methods to assess the effectiveness of individualized interventions contribute to disagreements about what constitutes evidence [15]. ABA has been widely acknowledged as the gold standard for treating ASD, supported by decades of research and a substantial body of corroborating evidence [16]. ABA is a popular and widely preferred method of treatment. The ranking or placement of therapies for ABA can vary based on several factors, such as the child's individual needs. Other treatments include speech, physical, occupational, nutritional, and cognitive behavioral therapy, play therapy, social skills training, and developmental approaches [15,16].

To a certain degree, findings on gender differences concerning the impacts of ABA on target behaviors with autistic individuals are limited and varied. ASD manifests differently, including variations in symptoms, severity, and co-occurring conditions. This heterogeneity can influence how an individual responds to ABA therapy. Considerations of culture and neurodiversity are crucial and can significantly influence research, practice, and discussions among stakeholder groups [17].

The wide array of procedures in ABA introduces complexity when analyzing the components and parameters that contribute to its effectiveness [18]. A study examined gender differences in core symptoms, associated features, and treatment response in a sample of 682 youth (585 males, 97 females) with ASD. The participants, aged between 3- 17 years (average age = 7.4 years), were part of six federally funded, multisite, randomized clinical trials. The study found no significant gender differences in the clinical characteristics of youth with ASD and their response to treatment [19].

It was found that gender predicted behavioral growth rates resulting from ABA interventions. Male participants tended to show faster improvement in adaptive behavior and physical development. Despite the significance of this finding, there was a notable discrepancy in the number of male (n=27) and female (n=8) participants (3.4:1) in this study. This study's limited number of female participants might have resulted in restricted variability. The researchers also noted that other studies did not find gender to be a predictor of treatment outcomes [20].

A group of researchers conducted an experimental study involving 100 children with autism. The experimental group comprised 50 young individuals, with an average age of 6.8 years (standard deviation = 1.2), including 30 males and 20 females. Like the experimental group, the control group included 50 young individuals with a marginally younger average age of 6.5 years (standard deviation = 1.5), comprising 35 males and 15 females. The researchers found that males had significantly lower scores on the Stereotyped Behavior Scale (SBS) than females (p=0.039). This finding suggested that evidence-based ABA treatments were more successful in reducing stereotypical behaviors among male participants compared to female participants [21].

Objectives

Much of the research does not explicitly address gender differences in the effectiveness of ABA treatment for ASD. More research is needed to definitively answer a research question on gender differences (or non) as the result of ABA treatments.

Considering the scarcity of research specifically investigating gender differences in response to ABA therapy in individuals with ASD, the goals of this replication study are: (i) to provide corroborative evidence for the 4:1 male-to-female ratio documented in scientific literature, and (ii) to determine the presence or absence of a gender difference by measuring general aggregate target behaviors across repeated measures time point intervals.

## Materials and methods

Study participants

Three to five behavioral technicians per child collected daily general target mastery data for 263 individuals with autism. This data was gathered using a large N design through retrospective chart reviews within the “Catalyst” tracking software (DataFinch Technologies, Atlanta, USA) [22]. Three separate datasets were collected from June 7, 2023 to January 7, 2024.

Dataset #1: June 7, 2023 to July 7, 2023 - n=98 (one month, three time points)

Dataset #2: July 7, 2023 to August 9, 2023 - n=103 (one month, three time points)

Dataset #3: August 9, 2023 to January 7, 2024 - n=62 (five months, 12 time points)

From June 7, 2023 to January 7, 2024, behavior analysts employed a mixed model of discrete trial training, mass trials, and naturalistic environment treatment over seven months. General target mastery data was assembled for 259 children and four adults, with seven data instances missing.

Inclusion/exclusion criteria

This study encompassed both males and females who were diagnosed with ASD by a psychiatrist, psychologist, or primary care physician. The participants ranged from one to 73 years old and were medically cleared for treatment.

Individuals who were not included in this study were those without an ASD diagnosis, those with a medical condition or disability that could render ABA therapy unsafe, those with a history of abuse, neglect, or trauma that could impede their ability to benefit from ABA therapy, those who were undergoing another intervention that was incompatible with ABA therapy, and cases where significant issues related to the treatment plan could not be resolved between the family and the provider.

Data collection procedures

Catalyst, a digital tool, helps behavioral therapists collect and analyze large volumes of behavioral data, replicating traditional paper-based methods. Board Certified Behavior Analysts (BCBAs) created individual treatment plans, incorporating behavior reduction and skill acquisition programs. Technicians assigned to specific individuals with autism used real-time data stamping to record behavior as it occurred. Using an iPad, they defined the problematic behavior and selected continuous measurement systems. This data was then available online for analysis and reporting.

Autistic individuals were treated at The Oxford Centers in Brighton and Troy, USA. These centers specialize in a mixed methods approach to ABA, using discrete trial training, mass trials, and naturalistic environment training. Each subject received a personalized treatment plan from one of eight BCBAs.

Each person was assigned to one of 85 behavioral technicians and worked with three to five over the weeks for seven months. Appropriate materials were selected and arranged in rooms for discrete trial training, mass trials, or a naturalistic setting for real-world interaction. Technicians rotated daily, providing four to seven hours of treatment, at least 25 hours weekly.

Technician teams collected specific behavioral and skill data, monitored progress, noted the reduction of prompts and reinforcements as the skill was mastered, and assessed skill generalization and maintenance. Data was entered into a handheld Catalyst database and updated daily in a central database.

Key performance metrics

The study’s dependent variable was the composite scores for cumulative mastered general behaviors, measured in three separate datasets ranging from June 7, 2023 through January 7, 2024. These behaviors, defined by BCBAs and behavioral technicians, included daily living skills like routines, organization, time management, eating, toileting, and hygiene.

Participants learned expressive communication skills, such as speaking, expanding vocabulary, improving conversation, greeting, responding, asking for help, and making requests. Receptive language skills like following directions and identifying requested stimuli were also emphasized.

Social skills training included turn-taking, sharing, assertiveness, peer interaction, and responding to new people. Community skills were practiced in real-world settings, including interactions in stores, money management, grocery shopping, restaurant ordering, speaking to police, safe walking, park safety, and stranger safety.

The study’s independent variable was time, measured with three distinct datasets covering seven months with data measurements every two weeks. Given the variation in treatment plans, the treatments generally involved discrete trial training, mass trial instruction, and naturalistic environment treatment. Strong, clear, and repetitive reinforcers were used to teach new behaviors.

Naturalistic teaching enhances skill generalization to everyday settings, making learning more enjoyable and encouraging engagement. This approach instills confidence in these procedures as an effective method for autism therapy. ABA interventionists teach responses, create contact with natural reinforcers, and allow the individual’s interests to guide teaching. Education is embedded within everyday activities in naturalistic environments, with some skills learned in a controlled setting before transitioning to a naturalistic one [23].

This study used a retrospective-repeated measures design in all three datasets [4,6,7], assessing the clinical application of ABA with functional analysis and discrete trial training in a naturalistic setting. The aim was to increase mastered target behaviors and decrease problematic behaviors over seven months. Repeated measures deal with outcomes measured on the same unit at different times or conditions, with each participant serving as their control [24,25].

Descriptive and inferential statistics

All statistical analyses, both descriptive and inferential, were conducted using the software SPSS version 29.0 (IBM Corp., Armonk, USA) [26]. The threshold for statistical significance, denoted by alpha (α), was set at 0.05. This implies that if the calculated p-values were less than 0.05, the null hypothesis would be rejected, signifying a statistically significant result.

A comprehensive summary of demographic information and baseline characteristics was compiled. This included generating summary frequency statistics for categorical variables such as gender and race/ethnicity and continuous variables like age and the respective time points for the three relevant datasets. The summary statistics encompassed central tendency and dispersion measures, including the data distribution's minimum and maximum, mean and standard deviation, and median.

To determine if there were statistically significant differences between male and female groups regarding the three datasets, three non-parametric Mann-Whitney U tests (Wilcoxen Rank Sum) were performed [27,28]. These tests compare the medians and distributions of two independent groups to determine whether there is statistical evidence that the associated population medians and distributions are significantly different. Each dataset was individually analyzed using non-parametric methods, which unlike parametric analysis do not necessitate the fulfillment of certain assumptions.

Interobserver reliability

Dataset #1

A two-way random effects model was applied, considering both the effects of individuals and measures as random variables. The intraclass correlation coefficient (ICC), specifically the two-way random effects model (2), was used. This model is typically employed when multiple measurements are taken from each rater and then averaged. The ICC (2) value was calculated to be 0.929 (with a 95% confidence interval of 0.831-0.964), indicating an excellent level of agreement among the raters. This value was larger than the average Pearson correlation coefficient of 0.892, suggesting that the ICC (2) was equally sensitive to the variability among raters and measurements. The Cronbach’s alpha for the three time point variables was r = 0.954, indicating a high internal consistency reliability [29,30].

Dataset #2

Likewise, a two-way random effects model was computed for this dataset, considering both the effects of individuals and measures as random variables. Again, the ICC (2) was used, which is typically employed when multiple measurements are taken from each rater and then averaged. The ICC (2) value was calculated to be 0.956 (with a 95% confidence interval of 0.931-0.972), indicating an excellent level of agreement among the raters. This value was more significant than the average Pearson correlation coefficient of 0.856, suggesting that the ICC (2) was equally sensitive to the variability among raters and measurements. The Cronbach’s alpha for the three time point variables was r = 0.974, indicating a high internal consistency reliability [29,30].

Dataset #3

Similar to the initial two datasets, a two-way random effects model was calculated, considering the effects of individuals and measures as random variables. Again, the ICC two-way random effects model (2) was utilized, typically employed when multiple measurements are taken from each rater and averaged. The ICC (2) value was found to be 0.860 (with a 95% confidence interval of 0.758-0.915), demonstrating an excellent level of agreement among the raters. This value was higher than the average Pearson correlation coefficient of 0.750, indicating that the ICC (2) was more sensitive to the variability among raters and measurements. The Cronbach’s alpha for the seven time point variables was r=0.910, signifying a high level of internal consistency reliability [29,30].

Institutional review board approval

This research was conducted retrospectively, utilizing data obtained from reviews of clinical charts. The study received an exemption (#1-1703366-1) from the WIRB-Copernicus Group (WCG) Institutional Review Board (IRB), signifying that it met the necessary ethical standards for research involving human subjects. The authors confirm that the research was conducted in accordance with the ethical principles outlined in the 1964 Declaration of Helsinki and its later amendments or equivalent ethical standards. These principles provide guidelines for conducting research involving human subjects, ensuring that participants' rights, safety, and well-being are protected. The ORC assigned the ClinicalTrials.gov Identifier: NCT06043284, has since rebranded itself as The Oxford Center (TOC). Additional identifiers for the study include OxRS-01-2021.

## Results

Dataset #1 - descriptive and inferential statistics

For the sample of 98 autistic children, the age was (M=9.0, SD=8.15), the median was 7.5, the minimum was one, and the maximum was 73. There were 70 males (71.4%) and 25 females (25.5%), with three (3.1%) missing values. There were 68 Caucasians (69.4%), 12 Asians (12.2%), five American Indian/Alaska Native (5.1%), four Hispanics (4.1%), and seven unspecified (7.1%), with two (2.0%) missing values. Regarding age categories, 17 (17.3%) were in the 1-4 years category, 37 (37.8%) were in the 5-8 years category, 20 (20.4%) were in the 9-12 years category, 12 (12.2%) were in the 13-16 years category and four (4.1%) were in the 17-73 years category, with eight (8.2%) missing values. Please note that four subjects were over 17 years old, e.g., 18 years old, 20 years old, 25 years old, and 73 years old. Table 1 displays the results of descriptive and inferential analyses covering the three repeated measures time points.

**Table 1:** Mann-Whitney U with confidence intervals and effect sizes for cumulative target behaviors

Outcome Measure	Gender	n	Mean	SD	Mean 95% CI (Lower)	Mean 95% CI (Upper)	Median	Median Difference	Median Difference 95% CI (Lower)	Median Difference 95% CI (Upper)	U	p-value	ή^2^ (Effect Size)
Targets Mastered Baseline	Male	68	5.26	8.68	3.16	7.37	3						
	Female	22	6.23	10.38	1.63	10.83	2	1	-2	2	727.5	0.846	0.0002
Targets Mastered 2 Weeks	Male	68	9.69	11.31	6.95	12.43	6						
	Female	22	11.05	14.17	4.76	17.33	6	0	-3	3	736	0.91	0.00005
Targets Mastered 4 Weeks	Male	68	11.21	11.82	8.34	14.07	7						
	Female	22	12.27	15.52	5.39	19.15	7	0	-3	4	687.5	0.569	0.001

Dataset #2 - descriptive and inferential statistics

For the sample of 103 autistic individuals, the age was (M = 9.23, SD = 7.94), the median was eight, the minimum was two, and the maximum was 73. There were seven missing values. There were 75 males (72.8%) and 27 females (26.2%), with one missing value. There were 75 Caucasians (72.8%), six Asians (5.8%), three Hispanics (2.9%), 12 Middle Eastern (11.7%), and seven African Americans (6.8%). There were no missing values. In terms of age categories, 18 (17.5%) were in the 1-4 years category, 37 (35.9%) were in the 5-8 years category, 22 (21.4%) were in the 9-12 years category, 15 (14.6%) were in the 13-16 years category, and four (3.9%) were in the 17-73 years category. There were seven (6.8%) missing values. Four subjects were over 17 years old, e.g., 18 years old, 20 years old, 26 years old, and 73 years old. Table 2 displays the results of descriptive and inferential analyses covering the three repeated measures time points.

**Table 2:** Mann-Whitney U with confidence intervals and effect sizes for cumulative target behaviors

Outcome Measure	Gender	n	Mean	SD	Mean 95% CI (Lower)	Mean 95% CI (Upper)	Median	Median Difference	Median Difference 95% CI (Lower)	Median Difference 95% CI (Upper)	U	p-value	ή^2 ^(Effect Size)
Targets Mastered Baseline	Male	68	16.67	14.09	13.25	20.07	11.5						
	Female	26	18.15	23.91	8.49	27.81	10.5	1.5	-3	8	781	0.383	0.003
Targets Mastered 2 Weeks	Male	68	20.50	17.07	16.37	24.63	16						
	Female	26	23.15	28.19	11.77	34.54	14.5	1.5	-5	8	819.5	0.585	0.001
Targets Mastered 4 Weeks	Male	68	25.71	20.49	20.74	30.67	17.5						
	Female	26	29.85	33.74	16.22	43.47	15.5	1.5	-7	10	825	0.618	0.001

Dataset #3 - descriptive and inferential statistics

For the sample of 62 autistic individuals, the age was (M=8.65, SD=4.53), the median was eight, the minimum was two, and the maximum was 26. There were 46 males (74.2%) and 14 females (22.6%), with two (3.2%) missing values. There were 34 Caucasians (54.8%), two Asians (3.2%), four Hispanics (6.5%), 16 Middle Easterns (25.8%), and four African Americans. There were two (3.2%) missing values. In terms of age categories, nine (14.5%) were in the 1-4 years category, 21 (33.9%) were in the 5-8 years category, 12 (19.4%) were in the 9-12 years category, seven (11.3%) were in the 13-16 years category, and two (3.2%) were in the 17-26 years category. There were 11 (17.7%) missing values. Two subjects were over 17 years old, e.g., 20 years old and 26 years old. Table 3 displays the results of descriptive and inferential analyses covering the 12 repeated measures time points.

**Table 3:** Mann-Whitney U with confidence intervals and effect sizes for cumulative target behaviors

Outcome Measure	Gender	n	Mean	SD	Mean 95% CI (Lower)	Mean 95% CI (Upper)	Median	Median Difference	Median Difference 95% CI (Lower)	Median Difference 95% CI (Upper)	U	p-value	ή^2 ^(Effect Size)
Targets Mastered Baseline	Male	46	8.15	9.39	5.36	10.94	6.5						
	Female	13	16.00	17.33	5.53	26.47	9	2.5	-9	2	395	0.198	0.007
Targets Mastered 2 Weeks	Male	46	11.00	11.75	7.51	14.49	9						
	Female	13	17.61	18.88	6.21	29.02	11	2	-11	4	373.5	0.365	0.003
Targets Mastered 4 Weeks	Male	46	12.93	19.62	9.11	16.76	12						
	Female	13	19.61	20.96	6.95	32.23	14	2	-12	5	363	0.471	0.002
Targets Mastered 6 Weeks	Male	46	15.59	13.92	11.45	19.72	13.5						
	Female	13	24.15	21.95	10.89	37.42	17	3.5	-14	5	366.5	0.436	0.003
Targets Mastered 8 Weeks	Male	46	21.07	17.43	15.89	26.24	17.5						
	Female	13	31.46	25.95	15.78	47.14	23	5.5	-19	6	371	0.391	0.003
Targets Mastered 10 Weeks	Male	46	27.26	18.53	21.76	32.77	25.5						
	Female	13	41.08	26.65	24.97	57.18	37	11.5	-22	5	394	0.208	0.007
Targets Mastered 12 Weeks	Male	46	33.46	22.56	26.76	40.16	29						
	Female	13	45.69	29.87	27.64	63.74	40	11	-23	9	373	0.373	0.003
Targets Mastered 14 Weeks	Male	46	36.72	24.74	29.37	44.06	33						
	Female	13	48.46	31.23	29.59	67.33	41	8	-24	11	371	0.387	0.003
Targets Mastered 16 Weeks	Male	46	39.13	26.16	31.36	46.89	35.5						
	Female	13	50.08	33.97	29.55	70.61	41	5.5	-23	13	464	0.512	0.002
Targets Mastered 18 Weeks	Male	46	40.09	26.57	32.19	47.98	37						
	Female	13	52.46	37.28	29.93	74.99	41	4	-25	13	356.5	0.546	0.002
Targets Mastered 20 Weeks	Male	46	40.41	27.08	32.37	48.45	37						
	Female	13	53.15	38.06	30.16	76.15	41	4	-25	14	357.5	0.535	0.002
Targets Mastered 22 Weeks	Male	46	40.41	27.08	32.37	48.45	37						
	Female	13	53.15	38.06	30.16	76.15	41	4	-28	11	350.5	0.346	0.004

## Discussion

This replication study had two main goals: (i) to substantiate the often-quoted 4:1 male-to-female ratio in scientific studies, and (ii) to ascertain if there is a significant difference between genders by assessing overall target behaviors with three separate datasets spaced two weeks apart following ABA interventions during seven months.

This study maintained the 4:1 ratio for male to female, as 74.9% of the analyzed sample was male and 25.1% were female [2,3]. Despite minor average gender differences, with females having slightly higher averages, using a non-parametric analysis, the study did not find any statistically significant differences between male and female individuals with autism across all three datasets, covering approximately seven months' worth of cumulative target behavior measurements. Effect sizes using Eta-squared (ή2) were very small for all three datasets.

This suggests that the ABA therapies used may have a similar effect on the subjects, irrespective of gender. However, it’s crucial to note that the wide confidence intervals reported in this study and the associated statistical uncertainty could imply considerable potential gender differences. Confidence intervals are values likely to encompass the actual population parameter. Wide intervals indicate a higher degree of uncertainty or variability in the data. This statistical uncertainty could potentially indicate gender differences.

The data might imply that the study’s results vary across genders, and there might be unique patterns or trends for different genders, which is reflected in the variability captured by the wide confidence intervals. This variability suggests that further research may be needed to fully understand the potential gender differences in response to ABA therapies.

Other researchers reported no significant differences between genders in their populations of infants/toddlers and children/adolescents in treatment response to ABA treatments. However, in the adult population, females showed a higher prevalence of social impairments (such as participation in social games, sports, activities, interest in the other person’s perspective during conversations, and imitation) and communication impairments (like interest in the other person’s perspective during conversations and understanding body language) compared to males [3].

Our study's average female scores were slightly higher across the seven time points, but these differences were not statistically significant. We also corroborated the 4:1 male-to-female ratio reported in the literature within our research subjects [31].

The absence of gender difference findings in the impact of ABA on individuals with autism could be attributed to several factors. Historically, autism research has predominantly focused on males due to the higher prevalence of autism in this gender. This bias has resulted in females and non-binary individuals often being neglected in studies, including those examining the effects of ABA. As a result, the impacts of ABA may not have been as extensively studied in females [32].

Females with autism often receive incorrect diagnoses or additional diagnoses such as bipolar disorder, depression, and anxiety. This complicates the evaluation of ABA effects and makes it challenging to identify gender differences. The underrepresentation of females in research further impedes the detection and analysis of gender differences in the impact of ABA [32].

The concept of neurodiversity underscores everyone's uniqueness, including those on the autism spectrum. As a result, the effects of ABA could significantly differ among individuals, irrespective of their gender. Autistic individuals possess unique insights into their own lives and communities. Therefore, the efficacy of ABA might be more influenced by individual variances rather than gender differences [33].

Autism is a spectrum disorder characterized by a broad range of symptoms and severity levels that can significantly differ among individuals. These individual variances can eclipse gender differences, making them challenging to detect and analyze in studies examining the effects of ABA treatments [12]. Despite the absence of gender differences in our present study, it’s crucial to acknowledge that individual responses to ABA treatments can vary extensively. Factors such as age, cognitive level, and severity of autism symptoms may affect treatment outcomes. Future research should delve deeper into these factors.

As previously mentioned, research has consistently shown a higher prevalence of ASD diagnoses in males than in females. This could potentially introduce a bias in the research data, with more males being represented in ABA studies than females [3,33]. Some studies have discovered that female individuals with ASD exhibited significantly better social interaction and social communication skills compared to male individuals with ASD. These inherent differences might influence the outcomes of ABA, making it challenging to compare the impacts of ABA between genders directly.

There have been criticisms of ABA from the autism caregiver community, which have significantly influenced research, practice, and discussions among stakeholder groups. These criticisms often pertain to the bias inherent in current practices, which have compromised the dignity and autonomy of many individuals with disabilities who have undergone ABA. This could potentially restrict the research on the impacts of ABA on different genders [31,33].

It’s important to acknowledge that while certain factors might restrict the availability of results on gender differences in the effects of ABA, they also underscore the necessity for more inclusive and personalized approaches in ABA and autism research. This study ventured into new territory by finding no statistically significant differences between male and female autistic individuals across all three datasets with cumulative target behavior time points measured every two weeks. These findings, accompanied by wide confidence intervals indicating a degree of uncertainty, suggest that ABA treatments may have similar effects on both males and females over time. This aligns with the principles of ABA that emphasize individualized and function-based strategies. The results highlight the importance of focusing on everyone's needs and characteristics rather than making gender-based assumptions.

There is an increasing awareness of the need for more inclusive research practices that consider gender differences in autism. This includes studies that outline gender differences in the effects of ABA treatments. The scarcity of studies outlining gender differences in the impact of ABA treatments on autistic individuals is likely due to a mix of historical bias, diagnostic complexities, underrepresentation of females in research, individual differences in autism, and the need for more inclusive research practices. Further research is needed to address these issues and enhance our understanding of gender differences in the effects of ABA treatments on autistic individuals.

This study adds to the limited research on gender differences in ABA treatment outcomes by demonstrating that ABA treatments may be equally effective for both genders, albeit with a degree of uncertainty. This study could provide valuable insights for clinicians, educators, and parents. The research advocates for the application of ABA treatments to all individuals with autism, regardless of their gender.

Limitations

This study does have certain constraints. The findings should be viewed with caution due to the limited number of studies in this field and several limitations in research that report non-statistically significant gender differences in individuals with autism. The large standard deviations calculated led to wide confidence intervals, indicating high variability or uncertainty in our data concerning a “true mean difference.”

Using a non-random sample may restrict the scope of this study, and the findings may not accurately represent the entire population of individuals with ASD or a broader context. There is no capacity to generalize beyond this sample. Furthermore, distinguishing differences between groups and fully accounting for potential confounding factors poses a challenge.

Several factors can influence this study’s outcomes, including the variability in task stimuli, the number of trials, the types of participants, the conditions of administration, and the focal task variable. These factors add complexity and make comparing results across different studies challenging.

Additionally, autism research was initially characterized and diagnosed primarily in boys and men, which may have resulted in an underrepresentation of females with smaller female samples, leading to a lower statistical power scenario relative to females. Research on gender differences, such as the present study, has focused on broad-ranging variables, which may not capture subtle differences inherent in the wide variation in ASD symptoms.

Misdiagnosis or additional diagnoses may occur in autistic individuals with co-occurring conditions such as anxiety, depression, and bipolar disorder. These may cloud the accuracy of measurements of treatment effects, making it difficult to determine gender differences.

These constraints underscore the necessity for more thorough and inclusive research methodologies in autism studies. It’s crucial to consider these factors when interpreting the outcomes of studies that report non-statistically significant gender differences in individuals with autism. While this study provides valuable insights, future research could address these limitations by employing a more varied and randomized sample, ensuring consistent administration conditions, and continuing the validation of the tools used. This approach would bolster the study’s robustness and increase the relevance of the findings.

Additional research is required to validate our findings and to investigate potential gender differences in other facets of ABA treatment, such as the involvement of parents and the intensity of treatment.

## Conclusions

The results of this study, which indicate non-significant gender differences in response to ABA treatments, may be of considerable interest. They suggest that ABA treatments could be equally beneficial for both males and females diagnosed with autism. However, these results should be interpreted prudently. It’s vital to highlight that the general pattern observed in this study, characterized by broad confidence intervals, carries a degree of statistical uncertainty that points towards potential substantial gender differences. Further research would be required to validate this hypothesis with an extension study and comprehend these possible differences. These findings may challenge existing assumptions about gender differences in treatment response. This could have significant implications for clinical practice, suggesting clinicians should not preferentially recommend ABA treatments for one gender over another. Instead, treatment recommendations should be based on the individual needs and characteristics of each child, irrespective of their gender. The researchers of this study hope these findings will stimulate further research in this area. Indeed, understanding the factors that influence treatment response is crucial for enhancing treatment outcomes and personalizing care. Future research could investigate other potential moderators of treatment response, such as age, severity of autism symptoms, or co-occurring mental health conditions. This could ultimately lead to more personalized and effective care for children diagnosed with autism.
